# Disturbing the Doxa of Patient Safety: Comment on "False Dawns and New Horizons in Patient Safety Research and Practice"

**DOI:** 10.15171/ijhpm.2018.26

**Published:** 2018-05-05

**Authors:** Joanne Travaglia

**Affiliations:** Centre for Health Services Management, Faculty of Health, University of Technology Sydney, Ultimo, NSW, Australia.

**Keywords:** Sociology of Safety, Sociology of Professions, Bourdieu

## Abstract

In a recent edition of this journal, Mannion and Braithwaite provide a succinct analysis of the emergence, and ultimately limited impact, of what they term the current ‘Safety I’ movement in healthcare. They describe the arc of this field from denial, through engagement via mechanisms and approaches imported from other industries, to the current situation where, despite ‘best efforts,’ error rates remain stubbornly recalcitrant. In examining the failure of system-wide efforts to produce sustained reductions in errors and adverse events, that article exposes the doxa, or what Bourdieu calls ‘the taken for granted’ which is central to this latest wave of patient safety movement. In this commentary, I would like to take focus on two key elements of Mannion and Braithwaite’s argument: that harm is caused by misguided but otherwise well-intentioned actions and the ‘embracing’ of patient safety. I then conclude by briefly considering the implications of these for Safety II, particularly as envisaged by the authors as an evolutionary, and therefore linear progression, from Safety I.

## Introduction

At the core of the current patient safety movement is the Socratic claim that no-one errs willingly.^1^ This assertion has provided a, if not the, foundational principal in what is presented as the transition from a socially mediated ‘shame and blame’ response to errors, to the current techno-rational systems approach.^2^ Attempts at prevention and remediation from this positioning have drawn heavily on industries such as aviation and mining, including the use of process controls, such a checklists.^3^ For Mannion and Braithwaite the importation of such ideas into healthcare are problematic because of cultural differences.^4^ I would argue, however, that the process of problematisation needs to go beyond the question of context. The positioning of the current patient safety movement as a progression from the ‘disciplining’^5^ of the individual to the disciplining of ‘the system’ operates to masks the continued impact of managerial and professional power relations.^6^ This includes the continuation of the blame and shame culture^7^ not just for those who err but, in numerous instances, those who attempt to prevent or mitigate the errors themselves.^8^

## The Notion of Misguided but Well-Intended Actions Causing Harm

Three separate issues arise from the argument that errors are the result of misguided but well-intentioned actions. First, the notion of ‘misguided’ asserts that individuals causing harm simply did not know better. The clinicians are said to be ‘doing their best’ so that errors when they arise, are not the result of an individual’s desire to harm, but rather a by-product of gaps in the system, Reason’s famous ‘swiss-cheese’ model.^9^

To somewhat torture the metaphor, what this model fails to address is the cheese around the gaps are as important as the gaps themselves. If the gaps allow the errors occur, then the cheese is what allows the gaps to occur. The persistence of errors, across countries and centuries, speaks to collective behaviour that is allowed to persist despite irrefutable evidence of significant harm and therefore seemingly beyond mechanistic solutions, current improvement strategies, all the way up to and including regulatory frameworks and convictions.^10^ Even given the multiple factors contributing to individual types of harm, their persistence despite all efforts would indicate the issue is not the gaps, but the cheese. One of the latest large-scale patient safety inquiries, that of the Mid Staffordshire NHS Foundation Trust, highlighted the lack of leadership and compassion,^11^ and not of knowledge, in the “… appalling standards of personal care and neglect at some hospitals and care home”^12^ (p.115).

The failure to gain significant improvements in hand hygiene rates^13^ is another case in point. Semmelweis,^14^ sought not only to avoid doing harm, but to prevent harm being done. The distinction is important. His personal experience of professional marginalization and abuse echoes in the present through the experience of whistle-blowers including Stephen Bolsin in the Bristol Royal Infirmary Inquiry^15^ and in many cases since.^8^

Second, the general claim of ‘well-intentioned’ interventions that unwittingly or unwillingly cause harm bears further critique. Medicine cannot and should not be allowed to distance itself from the large-scale iatrogenic harm caused to vulnerable individuals and populations in the past, and to date. The preeminent role of health professionals in torture and mass murder during WWII (within the living memory of survivors and with continued impact on their families),^16^ the Tuskegee (and various similar) ‘experiment(s)’ undertaken by clinicians and rationalized as being for ‘the greater good,’^17^ the actions of some clinicians in disaster settings,^18^ amongst many other systematic and sustained interventions which cause(d) harm for ideological reasons belies this ethically naive assumption. Similarly, arguments that these type of events are either mainly historical and or exceptional can be directly countered by the active participation of health care professionals in the sterilization of people with disabilities without their consent^19^ and in efforts to ‘convert’ the sexual orientation and identity of individuals^20^ which continue to date.

Third, unpacking the discourse around the well-meaning individual contrasted with the ‘bad apple’ not only highlights the use of ‘bad apples’ as scapegoats but exposes the mechanism of distancing the ‘good many’ in contrast to the lone (bad) individual. The ‘bad apple’ argument operates to reinforce the Socratic doxa.

There is clearly no doubt, for example, that Harold Shipman was a mass murderer, but his murders were conducted within the context of his daily medical practice. His actions were not hidden, they were unheeded. Early warning signs (such as the forging of prescriptions) were minimized or ignored by multiple colleagues and authorities, including the General Medical Council and the police.^21^ The social fact that his victims were virtually all elderly women, with limited voice and value, was largely ignored in the general debate around his actions.^22^ One of his reported rationales, that his victims were drains on the resources of the NHS, echoes the symbolic violence implicit in the continued descriptions of patients, and in particular elderly patients, as ‘bed blockers.’^23^ Indeed, as Harris notes, it was following the Shipman case that “… *medical educators began to argue that retaining the concept of altruism did a disservice to the medical profession where* ‘*it is the claim of altruism that allows the medical profession to claim moral superiority’*”^24^ (p. 3).

Shipman’s actions were extreme, but his reasoning, however utterly deluded, may have been drawn from standard health service discourse. Certainly, similar language pervades a variety of health, ageing and disability discourses that are in no way viewed as exceptional.

## Medical Professionals Have Embraced the Patient Safety Movement

Mannion and Braithwaite also make the argument that the medical profession had rejected, then accepted, and now embraced the patient safety agenda. An alternative interpretation of this trajectory is to see this ‘embracing’ as a mechanism for control of the patient safety agenda. From its emergence, the patient safety movement has largely been shaped by those whose perspectives are embedded within specific disciplines and power relations.^6^

The World Health Organisation’s first Patient Safety Curriculum Guide published in 2009,^25^ for example, was produced for medical schools. Given that there are more than double the number of nurses compared to doctors worldwide,^26^ the rationale was clearly not prioritised on the basis of workforce numbers. The second Curriculum Guide, published in 2011 was entitled ‘Multi-Professional,’ in other words ‘everyone else.’^27^

What then would patient safety agenda look like if the discourse of patient safety practice and education was led by patients, the public, survivors of errors, community mental health nurses, nutritionists, social workers or receptionists? A recent comparison of patient and regulators’ view on quality, for example, concluded that “*The predominant clinical approach taken by regulators does not match the patients’ perspective of what is relevant for healthcare quality … patients seem to be more tolerant of what they perceive to be clinical or management errors than of perceived relational deficiencies in care providers. If regulators want to give patients a voice, they should expand their horizon beyond the medical framework.”*^28^

## Can Safety II Disturb the Doxa?

Mannion and Braithwaite conclude their editorial by making an argument for an evolution from Safety I to Safety II.^29^ This approach is in line with a more general move across industries towards the use of positive psychology^30^ and appreciative inquiry^31^ as a way of addressing errors in organisations. There is no reason to believe, given the homeostasis achieved under Safety I, that Safety II could not provide fresh insights and new approaches to improving reducing errors and improving safety. To do so, however, it has to disrupt more than the doxa, to question not only what is done wrong compared to what is done right, or even linear versus complexity approaches, but will remain once this evolutionary leap has been achieved.

If Safety II is to succeed where Safety I failed, it needs to question not only the ontology, but the epistemology and ethics of the patient safety movement. Looking for successes over failures is only one part of the equation. The substantive human fault lines remain, whatever perspective is taken on the problem. Somewhere between the culpable and reckless individual and the unwilling agent of a deterministic culture lies the daily practice(s) of highly trained (including in patient safety) healthcare professionals.^32^ Yet as Dixon Woods has shown “*staff [do] not always do the right things, for a wide range of different reasons, including contestations about what counted as the right thing”*^33^ (p. 11). These are people whose practice is generated within culture(s) which continue to reward and punish, promote and supress. Bad apples are castigated, but so too are whistle-blowers, and while clinicians may not intend to cause errors, the system is constituted by people who individually and collectively make daily choices which can and do directly contribute to iatrogenic harm. In other words, in shifting the focus, Safety II must also change the doxa, directly exposing the assumptions, presumption and power relations which currently constitute the field of patient safety.

## Ethical issues

Not applicable.

## Competing interests

Author declares that she has no competing interests.

## Author’s contribution

JT is the single author of the paper.
